# Whole exome sequencing and the clinician: we need clinical skills and functional validation in variant filtering

**DOI:** 10.1007/s00415-015-7755-y

**Published:** 2015-05-10

**Authors:** Daniyal Daud, Helen Griffin, Konstantinos Douroudis, Stephanie Kleinle, Gail Eglon, Angela Pyle, Patrick F. Chinnery, Rita Horvath

**Affiliations:** Wellcome Trust Centre for Mitochondrial Research, Institute of Genetic Medicine, Newcastle University, Central Parkway, Newcastle upon Tyne, NE1 3BZ UK; Department of Medicine, James Cook University Hospital, Middlesbrough, TS4 3BW UK; Medical Genetic Center, 80336 Munich, Germany

**Keywords:** Genetics, Whole exome sequencing, Spastic paraplegia, Motor neuropathy

## Abstract

Whole exome sequencing (WES) is a recently developed technique in genetics research that attempts to identify causative mutations in complex, undiagnosed genetic conditions. Causative mutations are usually identified after filtering the hundreds of variants on WES from an individual’s DNA selected by the phenotype. We investigated a patient with a slowly progressive chronic axonal distal motor neuropathy and extrapyramidal syndrome using WES, in whom common genetic mutations had been excluded. Variant filtering identified potentially deleterious mutations in three known disease genes: *DCTN1*, *KIF5A* and *NEFH*, which have been all associated with similar clinical presentations of amyotrophic lateral sclerosis, Parkinsonism and/or hereditary spastic paraplegia. Predicting the functional effect of the mutations were analysed in parallel with detailed clinical investigations. This case highlights the difficulties and pitfalls of applying WES in patients with complex neurological diseases and serves as an instructive tale.

## Introduction

Whole exome sequencing (WES) is an increasingly available second-generation sequencing technique that identifies variants in the coding regions of the human genome [1]. In the field of neurology, it has helped patients by elucidating the cause of their often long-standing symptoms, and many previously undiagnosed patients with a range of neurological syndromes which can now be given the name of the causative gene and counselled appropriately. For researchers, WES has led to the rapid rise in the discovery of novel mutations in both known and novel disease genes, further unravelling the cellular function and relationships of various genes with human disease. However, one of the many limitations of WES is in its application to single patients with late-onset disease. WES of one individual’s DNA can identify up to 20,000 variants [1]. Even in patients with a characteristic clinical phenotype choosing the causative mutation remain a challenge if DNA from other affected or healthy relatives is not available to determine which variant segregates with the phenotype [2].

Here we describe a case wherein, we used WES to guide diagnosis in a patient with complex neurodegenerative disease.

## Methods

We assessed the patient’s clinical history, examination and serological, neurophysiological, radiological and histological investigations. Exome sequencing was carried out using genomic blood DNA by Illumina Truseq™ 62 Mb exome capture. We used our in-house bioinformatics pipeline to align data to the reference human genome (UCSC hg19), remove duplicate sequence reads (Picard v1.85) and variant detection (Varscan v2.29, Dindel v1.0110). Results were filtered for variants with a minor allele frequency less than 0.01 in several databases: dbSNP135, 1000 genomes (February 2012 data release), the National Heart, Lung and Blood Institute (NHLBI, NIH, Bethesda, MD) Exome Sequencing Project (ESP) 6500 exomes, and 238 unrelated in-house controls. Rare homozygous and compound heterozygous variants were defined, and protein altering and/or putative ‘disease-causing’ mutations, along with their functional annotation, were identified using ANNOVAR. Further filtering has been performed by gene ontology (GO) terms associated with neuronal function, and Online Mendelian Inheritance in Man (OMIM) disease descriptions related to neuropathy, ataxia or extrapyramidal disorders (i.e., the patient’s clinical phenotype). We carried out PCR (IMMOLASE™ DNA Polymerase, Bioline UK) and Sanger sequencing (BigDye^®^ Terminator v3.1) of variants predicted to be deleterious by five online prediction tools (MutationTaster, SIFT, Polyphen2, A-GVGD, and LRT).

## Results

### Case report

A 74-year-old left-handed man originally presented at the age of 45 with right-sided back pain and sciatica as well as bilateral grip weakness. His gait had also changed but he was able to walk and play sports. Past medical history included poliomyelitis at the age of ten, during the 1950s epidemic.

Both of his parents died at age 88 and 72, and had no neuromuscular symptoms. He has one son and four daughters (28–36 years of age), and one daughter was recently diagnosed with multiple sclerosis. He has a 71-year-old healthy brother who was not available for testing.

**Fig. 1:**
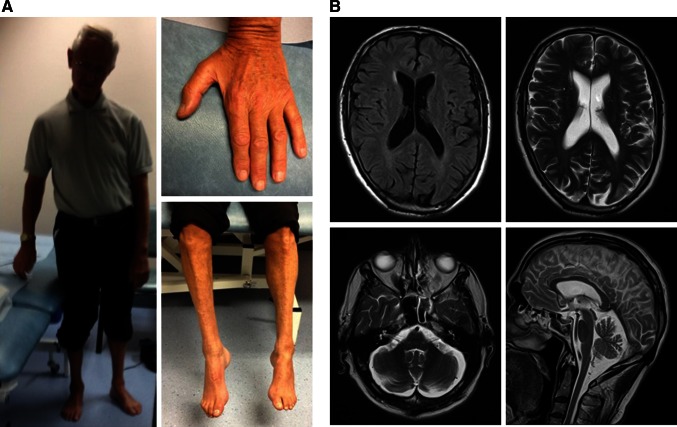
**a** Photographs of the patient demonstrating a partially treated torticollis, a broad-based stance, distal upper and lower limb atrophy as well as bilateral pes cavus. **b** MRI of the patient’s brain demonstrating cerebellar atrophy

At age 45, examination revealed wasting and weakness of the right sternocleidomastoid muscle, bilateral wasting of thenar and intrinsic hand muscles (Fig. 1a). In particular, the abductor pollicis brevis was weak bilaterally. There was distal lower limb weakness with foot drop but no pes cavus. Tone and reflexes were normal.

He was seen again at the age of 64 with worsening torticollis. He had jerky ocular pursuit movements and bilateral wasting of the tongue. His previous weakness had persisted with additional weakness in the shoulders. He had developed pes cavus bilaterally (Fig. 1a), brisk upper limb and knee reflexes with absent ankle reflexes bilaterally. He progressed to develop dysmetria and dysdiadochokinesis but no dysarthria.

More recent examination at 74 years revealed progression of the complex movement disorder with facial dystonia, head tremor, distal amyotrophy, ataxia and some rigidity at the wrists. He had a wide-based steppage gait with reduced arm swing.

### Investigations

Basic haematological and biochemical investigations were normal except for gamma-glutamyl transferase (83 units/L, normal <70), creatine kinase (193 and 215 U/L, normal 10–190), random glucose (8.3 mmol/L), cholesterol (7.9 mmol/L, normal <5.2) and triglycerides (6.1 mmol/L, normal fasting level <1.8). Thyroid function tests, auto-antibodies, Vitamin E, 24-h urinary copper, alpha-fetoprotein, lysosomal enzymes, hexosaminidase A were all within normal limits.

Brain MRI revealed marked cerebellar atrophy and mild cortical atrophy without signal change (Fig. 1b). Cervical MRI showed mild spondylotic changes at C5/6 and C6/7. DatScan showed no abnormalities in dopamine metabolism. Nerve conduction studies demonstrated preserved sensory nerve action potentials, sensory and motor conduction velocities but symmetrically diminished compound motor potentials in all four limbs, suggesting that the syndrome could not be attributed to previous poliomyelitis. Electromyography revealed chronic neurogenic changes, suggesting spinal muscular atrophy or distal spinal muscular atrophy. With electromyography, unstable polyphasic units were observed with reduced interference and amplitudes up to 10 mV, confirming a chronic progressive anterior horn cell disease. Muscle biopsy showed only neurogenic changes.

### Genetic analysis

Genetic tests performed before WES excluded Huntington’s disease (*IT15*), spinal muscular atrophy (*SMN1*), spinal bulbar muscular atrophy (AR), primary torsion dystonia (*DYT1*), and the common forms of spastic paraplegias (*SPG4, REEP1, ATL1, SPG7*). No mtDNA deletions were detected in his muscle DNA. Rare, potentially disease-causing variants found in filtered WES data are listed in Tables 1 and 2; we could confirm all variants in heterozygous state in the patient’s DNA by Sanger sequencing. No further mutations were detected in any of the known disease genes involved in inherited movement disorders, neuropathies or spastic paraplegias and no further family members were available for genetic analysis.

**Table 1:** Predicted deleterious variants filtered from whole exome sequencing data

Gene	Position	Variant	Bioinformatic score	Disease association
DCTN1	Chr2:g.74588640 C	NM_004082.4: c.3823 C > T, p.Arg1275Cys	5	ALS, Perry syndrome
KIF5A	Chr12:g.57965854 C	NM_004984.2: c.1373 C > T, p.Ser458Phe	3.5	HSP, CMT
NEFH	Chr22:g.29885580_2988 603	NM_021076.3: c.1965 1988del, p.Glu658_Lys665del	N/A	ALS

Scores were calculated from the output of five mutation deleteriousness prediction programmes: SIFT (score 1 for prediction of ‘Deleterious’, 0 for others), LRT (score 1 for prediction of ‘Deleterious’), Polyphen2 (score 1 for ‘probably damaging’ prediction, 0.5 for ‘possibly damaging’ prediction, 0 for ‘benign’), Align-GVGD (score 1 for predictions C55 or C66, score 0 for other predictions) and MutationTaster (score 1 for ‘disease-causing’ prediction, 0 for ‘polymorphism). Predictions were not available for the deletion in the NEFH gene

**Table 2:** Coverage and depth statistics of exome sequencing for the patient

Mean target base coverage	Number of bases covered 20-fold	% Bases covered 20-fold	Number of bases covered tenfold	% Bases covered tenfold	Number of bases covered fivefold	% Bases covered fivefold	Number of bases covered onefold	% Bases covered onefold
88.23	29129128	91.21	30151406	94.41	30585401	95.77	31131999	97.49

Coverage calculated for Consensus Coding Sequence (CCDS) bases (31,935,069 bp)

## Discussion

We have found novel, potentially pathogenic mutations in the patient in multiple genes (*DCTN1*, *KIF5A*, *NEFH*) associated with various neurological syndromes. Based on previous reports, heterozygous mutations in all three genes can be associated with similar clinical presentation observed in our case. In further support of the pathogenic role, the selected variants have not been reported previously at all (*KIF5A*) or are extremely rare (one single heterozygous *DCTN1* and *NEFH* variant in 65,000 exomes (http://exac.broadinstitute.org/).

*DCTN1* mutations have been associated with a familial form of ALS (p.Gly59Ser) [3], familial progressive supranuclear palsy [4], Perry syndrome (p.Gly71Ala/Glu/Arg, p.Thr72Pro, and p.Gln74Pro) which is characterized by central hypoventilation and parkinsonism [5]. Our patient exhibited a distal motor neuropathy with ataxia and an extrapyramidal syndrome of tremor and focal dystonia—an inexact fit with the previously described phenotypes of *DCTN1*. However, the involvement of the spinal motor neurones in combination with a movement disorder resembles *DCTN1*-related disease and our patient may represent a new phenotype associated with *DCTN1* mutation.

*DCTN1* codes for the p150^glued^ subunit of the dynactin complex, which together with dynein is responsible for retrograde transport of vesicle cargo in axons [6]. The C-terminal region of p150^glued^ has been shown in *N. crassa* to be responsible for the interaction of the dynactin complex with membranous cargo [7]. However, previous work in HeLA and COS7 cells could not confirm that mutations in the C-terminal region of DCTN1 (Arg1101Lys and Thr1249Ile) are functionally pathogenic [8]. Our patient’s *DCTN1* mutation is located to this region; nevertheless, this variant has not been reported previously and no functional data are available on it to date. However, functional in vivo imaging studies in patients possessing the variants associated with Perry syndrome, have demonstrated a reduced uptake of FDOPA, a marker of dopamine synthesis and storage [9].

Mutations in *KIF5A* are associated with spastic paraplegia (*SPG10*), and can also cause peripheral neuropathy, parkinsonism, retinitis pigmentosa and behavioural problems [10, 11]. Therefore, the pyramidal signs in our patient may also represent a *KIF5A*-related disease. *KIF5A* codes for the kinesin heavy chain which is responsible for anterograde transport of cargo (thought to be neurofilaments) towards the synapse [12]. The *KIF5A* mutation in our patient occurs in the neck region that links two domains in the protein [10]. To our knowledge, only one mutation (p. Ala361Val) has previously been reported in the neck region in spastic paraplegia [13]; however, its pathogenic role has not been confirmed in functional studies [14].

WES also detected a heterozygous 24-base pair deletion in the neurofilament heavy chain (*NEFH*) gene in our patient. Such deletions of a lysine-serine-proline unit, that shorten the repetitive tail region of the NEFH protein, have been associated with an increased risk of developing ALS [15], but no highly penetrant causative alleles have been described in this gene.

Another intriguing point is the poliomyelitis in our patient’s previous history. Whether the childhood neuropathy was indeed poliomyelitis, and coupled with a potentially deleterious mutation contributed to the development of a slowly progressive predominantly distal motor neuropathy, or the childhood onset polio-like neuropathy represents the first manifestation of a genetic condition remains unclear.

This case illustrates the problems that can be encountered in using next generation sequencing to guide the diagnosis of sporadic cases. Despite having confirmed potentially disease-causing mutations in *DCTN1* and *KIF5A*, as well as a genetic predisposing variant in *NEFH*, we cannot surely prove that they are the cause for the patient’s phenotype without an informative family history, which is often not available in late-onset disease. The absence of clinical signs and symptoms in younger family members may simply reflect a later disease onset or low penetrance of the mutation rather than its non-pathogenicity. Moreover, we cannot exclude the possibility that more than one of the variants are relevant, and the different aspects of the patient’s condition potentially may have independent causes.

The case also highlights the importance of clinical history and examination in filtering through the enormous number of possible diagnoses generated by the results of next generation sequencing, and the need for functional studies at a molecular level to identify which mutation contributes to the cellular dysfunction. The overlapping phenotypes of genetic forms of ALS, HSP and dystonia highlight the importance of an unbiased testing approach, but also illustrate the difficulties of confirming the genetic diagnosis and functional tests provided evidence for the pathogenicity of these mutations are still lacking.

In conclusion, we present a patient with a complex neurological phenotype including predominantly distal motor neuropathy, extrapyramidal and cerebellar signs for whom we have identified a number of potentially pathogenic candidate mutations.

## Abbreviations

ALS: Amyotrophic lateral sclerosis
HSP: Hereditary spastic paraplegia
CMT: Charcot–Marie–Tooth disease
